# Supplementary material for: Determining the Minimally Effective Dose of a Clinical Candidate AAV Vector in a Mouse Model of Crigler-Najjar Syndrome
Source: Mol Ther Methods Clin Dev. 2018 Jul 21;10:237–44. doi: 10.1016/j.omtm.2018.07.008 (PMC6090885; doi:10.1016/j.omtm.2018.07.008)
Supplement: Document S1. Tables S1–S3 [file mmc1.pdf]

OMTM, Volume *10*

Supplemental Information

Determining the Minimally Effective Dose of a Clinical Candidate AAV Vector in a Mouse Model of Crigler-Najjar Syndrome

Jenny A. Greig, Jayme M.L. Nordin, Christine Draper, Deirdre McMenamin, Edward A. Chroscinski, Peter Bell, John T. Gray, Laura K. Richman, and James M. Wilson

Supplemental Table 1. Composition of study groups.

Dose	Female		Male	
	Mouse	Age (weeks)	Mouse ID	Age (weeks)
$2.5x10^{10}$ GC/kg	5228	8	4093	12
	6944	11	4913	8
	7524	6	4971	7
	6851	11	5018	7
	6678	15	5020	7
$2.5x10^{11}$ GC/kg	7426	7	5147	7
	6311	18	5149	7
	6753	13	5182	9
	6211	20	5264	7
	7442	7	6989	9
$2.5x10^{12}$ GC/kg	5054	8	4161	7
	5127	7	4162	7
	5128	7	4178	7
	5129	7	4553	6
	5198	9	4573	6
$2.5x10^{13}$ GC/kg	4158	6	4094	7
	4159	6	2786	13
	988	11	2764	15
	2782	13	2717	15
	2374	19	4576	6
Vehicle control	983	11	2785	13
	2369	19	2716	15
	2375	19	990	11
	4044	8	2718	15
	4549	6	945	11

Supplemental Table 2. Histopathology summary from vector- and vehicle control-injected UGT1 KO mice.

**Cohort**	**1**	**1**	**2**	**2**	**3**	**3**	**4**	**4**	**5**	**5**
**Sex**	**M**	**F**	**M**	**F**	**F**	**M**	**F**	**M**	**F**	**M**
**No. of animals evaluated per cohort**	**5***	**5***	**6***	**5***	**5***	**5***	**5**	**5**	**5**	**5**
**Liver**										
No. examined	5	5	6	5	5	5	5	5	5	5
No abnormalities detected	3	2	5	-	3	2	4	2	5	4
	---	---	---	---	---	---	---	---	---	---
Single cell hepatocellular necrosis/degeneration, centrilobular										
Grade 1	2	2	1	4	2	3	1	1	-	1
Grade 2	-	-	-	-	-	-	-	2	-	-
	---	---	---	---	---	---	---	---	---	---
Mononuclear cell infiltration										
Grade 1	-	1	1	5	2	-	1	3	-	-
Grade 2	-	1	-	-	-	-	-	-	-	-
	---	---	---	---	---	---	---	---	---	---
Mitotic figures, hepatocellular										
Grade 1	-	-	-	1	1	-	-	1	-	-
	---	---	---	---	---	---	---	---	---	---
Bile stasis										
Grade 1	-	-	-	-	1	-	-	2	-	1
**Lung**										
No. examined	-	-	-	-	-	-	5	5	5	5
No abnormalities detected	-	-	-	-	-	-	5	5	3	3
	---	---	---	---	---	---	---	---	---	---
Alveolar macrophages, acidophilic material accumulation										
Grade 1	-	-	-	-	-	-	-	-	1	1
Grade 2	-	-	-	-	-	-	-	-	-	1
	---	---	---	---	---	---	---	---	---	---
Infiltrate, subacute, focal										
Grade 1	-	-	-	-	-	-	-	-	1	-
**Uterus**										
No. examined	-	-	-	-	-	-	5	-	5	-
No abnormalities detected	-	-	-	-	-	-	5	-	4	-
	---	---	---	---	---	---	---	---	---	---
Inflammation, subacute, focal										
Grade 1	-	-	-	-	-	-	-	-	1	-
**Skin, injection site**										
No. examined	-	-	-	-	-	-	5	5	5	5
No abnormalities detected	-	-	-	-	-	-	4	5	5	5
	---	---	---	---	---	---	---	---	---	---
Dermal fibrosis										
Grade 2	-	-	-	-	-	-	1	-	-	-

Supplemental Table 3. Tissues harvested for histopathology.

Tissues harvested for histopathology		
Adrenal glands	Jejunum	Skeletal muscle
Brain	Kidneys	Spleen
Cecum	Liver	Sternum
Colon	Lungs	Stomach
Duodenum	Mandibular lymph node	Testes
Epididymides	Mesenteric lymph node	Thymus
Eyes	Ovaries	Thyroids with parathyroids
Heart	Pancreas	Urinary bladder
Ileum	Salivary glands	Uterus
Injection site	Sciatic nerve	Gross lesions (if any)
