# Supplementary material for: Determining the Minimally Effective Dose of a Clinical Candidate AAV Vector in a Mouse Model of Crigler-Najjar Syndrome
Source: Mol Ther Methods Clin Dev. 2018 Jul 21;10:237–44. doi: 10.1016/j.omtm.2018.07.008 (PMC6090885; doi:10.1016/j.omtm.2018.07.008)
Supplement: Document S2. Article plus Supplemental Information [file mmc2.pdf]

Molecular Therapy
Methods & Clinical Development
Original Article

AMERICAN SOCIETY of GENE & CELL THERAPY

Determining the Minimally Effective Dose of a Clinical Candidate AAV Vector in a Mouse Model of Crigler-Najjar Syndrome

Jenny A. Greig,[1] Jayme M.L. Nordin,[1] Christine Draper,[1] Deirdre McMenamin,[1] Edward A. Chroscinski,[1] Peter Bell,[1] John T. Gray,[2] Laura K. Richman,[1] and James M. Wilson[1]

[1]Gene Therapy Program, Department of Medicine, Perelman School of Medicine, University of Pennsylvania, Philadelphia, PA, USA; [2]Audentes Therapeutics, San Francisco, CA, USA

Liver metabolism disorders are attractive targets for gene therapy, because low vector doses can reverse the buildup of toxic metabolites in the blood. Crigler-Najjar syndrome is an inherited disorder of bilirubin metabolism that is caused by the absence of uridine diphosphate glucuronosyl transferase 1A1 (UGT1A1) activity. This syndrome is characterized by hyperbilirubinemia and jaundice. Unfortunately, current phototherapy treatment is not effective long term. We intravenously injected phototherapy-rescued adult UGT1 knockout mice with 2.5 $\times$ 10^{10}–2.5 $\times$ 10^{13} genome copies (GC)/kg of a clinical candidate vector, AAV8.TBG.hUGT1A1co, to study the treatment of disease compared to vehicle-only control mice. There were no apparent vector-related laboratory or clinical sequelae; the only abnormalities in clinical pathology were elevations in liver transaminases, primarily in male mice at the highest vector dose. Minimal to mild histopathological findings were present in control and vector-administered male mice. At vector doses greater than 2.5 $\times$ 10^{11} GC/kg, we observed a reversal of total bilirubin levels to wild-type levels. Based on a significant reduction in serum total bilirubin levels, we determined the minimally effective dose in this mouse model of Crigler-Najjar syndrome to be 2.5 $\times$ 10^{11} GC/kg.

INTRODUCTION

Monogenic disorders of liver metabolism are attractive targets for gene therapy applications because the buildup of toxic metabolites in the blood and other tissues potentially can be reversed at low vector doses by the transduction of a few hepatocytes. Crigler-Najjar syndrome is an ultra-rare autosomal recessive disorder of bilirubin metabolism, characterized by hyperbilirubinemia and jaundice. Patients with Crigler-Najjar syndrome type 1 lack the enzyme uridine diphosphate glucuronosyl transferase 1A1 (UGT1A1), which is responsible for the conjugation of bilirubin for excretion. Without intervention, the resulting elevated blood bilirubin levels can lead to irreversible neurological damage. Current treatment for Crigler-Najjar syndrome includes an average of 12 hr per day of phototherapy. However, most patients eventually require a liver transplant because phototherapy becomes less effective with age.

A potential approach to treating Crigler-Najjar syndrome is systemic delivery of a gene therapy vector expressing UGT1A1, which would enable the liver to continuously synthesize UGT1A1. The aim of the current study was to determine the minimally effective dose (MED) of a clinical candidate vector, AAV8.TBG.hUGT1A1co, before clinical trials. We have successfully used mouse models of specific human liver diseases to determine the MED and toxicity of vectors.[1,2] Two UGT1 knockout (KO) mouse strains are phenotypically similar to patients with Crigler-Najjar syndrome.[3,4] One strain contains the same point mutation as the Gunn rat, and the other has a neomycin cassette inserted into the *Ugt1* gene locus.[3–6] Both UGT1 KO mouse models display lethal hyperbilirubinemia in the immediate postnatal period, and mice do not survive past day 11 of life without intervention. Phototherapy via exposure to blue fluorescent light for 12 hr per day for the first 21 days of life has been shown by us and others to allow the UGT1 KO mice to survive until adulthood and exhibit serum total bilirubin levels of 9.1 ± 3.0 mg/dL following weaning from phototherapy.[4,7–10] Thus, pretreatment with phototherapy allows the administration of gene therapy vectors to be delayed until adulthood, which is after the most proliferative phase of liver development, increasing the likelihood of stable, long-term gene expression. Therefore, we intravenously (i.v.) injected phototherapy-rescued adult UGT1 KO mice with a clinical candidate vector, AAV8.TBG.hUGT1A1co. We evaluated levels of serum total bilirubin and liver transaminases and performed a comprehensive histopathological examination to determine vector efficacy and the MED to support a future clinical trial.

RESULTS

Rationale and Design

The UGT1 KO mouse is a disease model for Crigler-Najjar syndrome. As this disease can affect both sexes in the human population, we used

Received 7 February 2018; accepted 17 July 2018;
https://doi.org/10.1016/j.omtm.2018.07.008.

Correspondence: James M. Wilson, MD, PhD, Gene Therapy Program, Department of Medicine, Perelman School of Medicine, University of Pennsylvania, 125 South 31st Street, Suite 1200, Philadelphia, PA 19104, USA.
E-mail: wilsonjm@upenn.edu

Check for updates

This is an open access article under the CC BY license (http://creativecommons.org/licenses/by/4.0/).

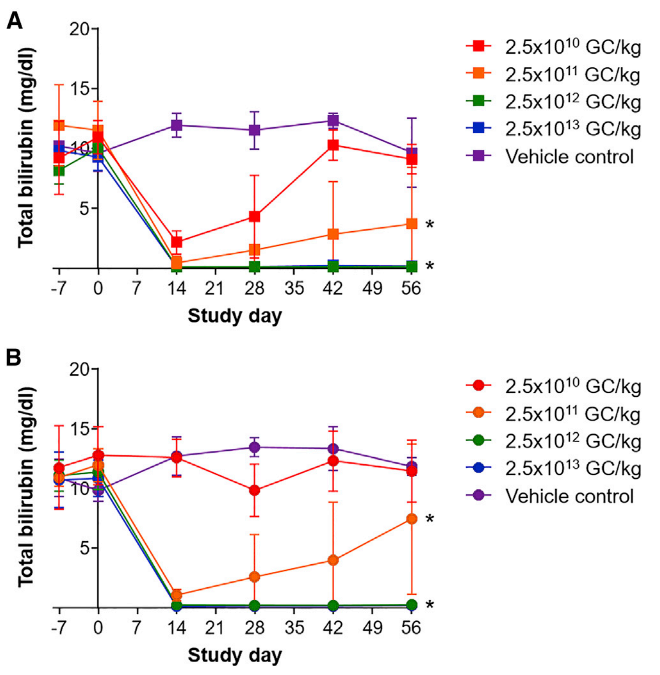

Figure 1. Serum Total Bilirubin Levels in Vector- or Vehicle Control-Injected UGT1 KO Mice

Male (A) and female (B) UGT1 KO mice received an i.v. injection of 2.5×10^{10}, 2.5×10^{11}, 2.5×10^{12}, or 2.5×10^{13} GC/kg of AAV8.TBG.hUGT1A1co or vehicle control. Total bilirubin levels were measured in serum samples. Values are expressed as mean ± SD. *$p < 0.05$.

both male and female UGT1 KO mice to determine the MED. We exposed all UGT1 KO mice to phototherapy immediately after birth for 12 hr per day for up to 21 days (blue fluorescent light, $\lambda = 450$ nm; 10–30 $\mu W/cm^2/nm$). Male and female UGT1 KO mice aged 6–20 weeks with a body weight of more than 15 g received an i.v. tail vein injection of vehicle control or AAV8.TBG.hUGT1A1co (AAV8 viral capsid with thyroxine binding globulin [TBG] promoter and a codon-optimized version of human UGT1A1 [hUGT1A1co]) at one of four doses: 2.5×10^{10}, 2.5×10^{11}, 2.5×10^{12}, or 2.5×10^{13} genome copies (GC)/kg (n = 5/sex/group). The composition of the groups is shown in Table S1. We performed necropsies on all mice 56 days after administration to capture hUGT1A1 expression profiles and detect any potential toxicity against the transgene.

Clinical Findings

During the course of the study, we euthanized one mouse for humane reasons. The mouse had received a dose of 2.5×10^{11} GC/kg and showed a moribund condition on day 8 after administration. Following a full necropsy, we collected tissue samples and performed histopathological analyses. The microscopic findings for this mouse were moderate extramedullary hematopoiesis in the spleen and marked apoptosis of thymic lymphocytes, which is a background finding related to stress. As this mouse was euthanized at such an early stage in the study, we enrolled an additional male mouse into the same cohort to provide a complete dataset.

Reversal to Wild-Type Total Bilirubin Levels

The contract facility Antech Good Laboratory Practice (GLP) measured the serum total bilirubin levels of all animals before vector administration and throughout the study. UGT1 KO mice injected with vehicle control showed similar total bilirubin levels across all time points with no sex-specific variation (Figure 1A for males; Figure 1B for females). Elevated total bilirubin levels rapidly reversed to normal wild-type baseline levels (0.1–0.3 mg/dL)[10] by day 14 after vector administration at two doses (2.5×10^{12} and 2.5×10^{13} GC/kg) in both males and females. Male mice administered with 2.5×10^{11} GC/kg showed significantly reduced serum total bilirubin levels, reaching 0.5 ± 0.5 mg/dL at day 14 (mean ± SD, Wilcoxon rank-sum test, $p < 0.05$). By contrast, female mice administered with the same dose of vector (2.5×10^{11} GC/kg) had serum total bilirubin levels of 1.0 ± 0.5 mg/dL at day 14 (mean ± SD). This reduction in bilirubin was transient in some mice administered with 2.5×10^{11} GC/kg, with two males and three females showing rising levels after day 14 after vector administration. In male mice, administration of the 2.5×10^{10} GC/kg dose reduced serum total bilirubin levels by 79% at day 14 after vector administration; these levels gradually increased to 57% at day 28 and returned to baseline hyperbilirubinemia by day 42 (Figure 1A). Administration of the 2.5×10^{10} GC/kg dose in female UGT1 KO mice did not change serum total bilirubin levels from baseline values. We compared total bilirubin levels across the groups using linear mixed-effect modeling and stratified the analysis by sex. We observed a significant reduction in total bilirubin compared to the respective control group for all dose groups with the exception of female mice administered with 2.5×10^{10} GC/kg ($p < 0.05$).

Dose-Dependent Elevations in Transaminases

Antech GLP also analyzed blood chemistries throughout the in-life phase of the study and at the time of necropsy. We evaluated alanine transaminase (ALT) (Figure 2A) and aspartate transaminase (AST) (Figure 2B) values for male and female mice after vector administration and compared them to control mice (e.g., the average value for day 28 vector-administered mice was compared to the average value for day 28 control male mice). Transaminase abnormalities were primarily present in a dose-dependent manner on day 28 after vector administration in males that received the highest vector dose. Only small changes in ALT or AST levels were present among mice that received lower vector doses. Elevations in ALT greater than 4-fold above baseline levels were restricted to the highest dose group (2.5×10^{13} GC/kg), with the exception of one male mouse in the lowest dose group (2.5×10^{10} GC/kg) at day 14 and one male mouse that received a dose of 2.5×10^{11} GC/kg at day 28 (Figure 2A). Elevations in AST showed a similar pattern but were less than 4-fold above baseline levels, with the exception of one female that received a dose of 2.5×10^{12} GC/kg at day 56 (Figure 2B).

We compared ALT and AST levels across the cohorts using linear mixed-effect modeling, stratified by sex. ALT was significantly elevated compared to the control group in both male and female mice administered with the highest vector dose ($p = 0.015$ for males;

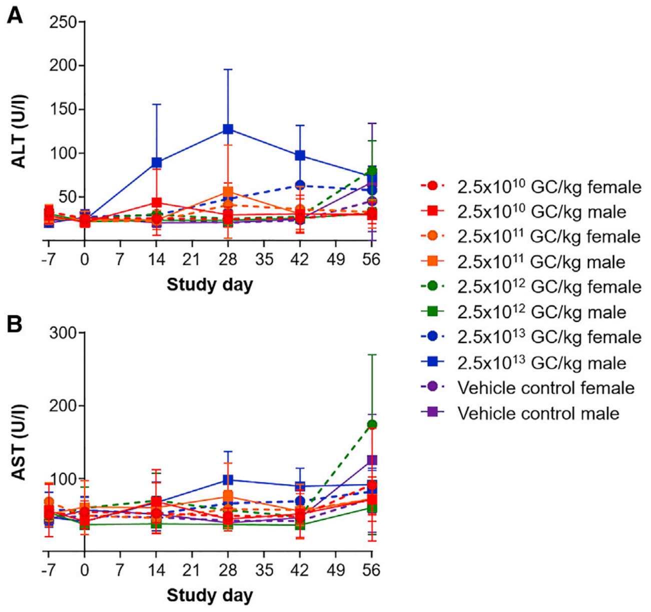

Figure 2. ALT and AST Levels in Vector- or Vehicle Control-Injected UGT1 KO Mice

Male and female UGT1 KO mice received an i.v. injection of 2.5×10^{10}, 2.5×10^{11}, 2.5×10^{12}, or 2.5×10^{13} GC/kg of AAV8.TBG.hUGT1A1co or vehicle control. (A) ALT and (B) AST levels were measured in serum samples. Values are expressed as mean ± SD.

p = 0.049 for females). By contrast, AST was significantly elevated compared to the control group only in female mice administered with the highest vector dose (p = 0.042). However, there was no correlation between the age of mice at the time of vector administration and transaminase elevation ($R^2 = 0.026$ for ALT and $R^2 = 0.0005$ for AST at day 28).

Histopathology Findings

We harvested tissues from all animals at the time of necropsy and stained them with H&E for a full histopathology analysis. An experienced board-certified veterinary pathologist microscopically examined sections from all harvested tissues in a blinded manner for the highest vector dose group and the vehicle control group. Liver sections were evaluated for all dose groups (Table 1). Gross observations were noted in a few animals, including an enlarged mandibular lymph node in a male administered with 2.5×10^{12} GC/kg of vector, a dilated uterus in a female administered with 2.5×10^{13} GC/kg, and a dilated uterus and slight icterus in a female administered with vehicle control.

Microscopic findings were predominantly found in the liver and characterized by centrilobular single-cell hepatocellular necrosis and/or degeneration and mononuclear cell infiltration (Table 1). Observations of centrilobular single-cell hepatocellular necrosis and/or degeneration were minimal in all female mice and most male mice. However, we observed mild centrilobular single-cell hepatocellular necrosis and/or degeneration in two male mice administered with 2.5×10^{13} GC/kg. Three male mice that received the highest vector dose displayed minimal mononuclear cell infiltration, which was more prevalent in female mice; one female mouse administered with 2.5×10^{10} GC/kg showed mild mononuclear cell infiltration. Other findings in the liver included minimal hepatocellular mitotic figures in one female dosed with 2.5×10^{11} GC/kg, and minimal hepatocellular mitotic figures and minimal bile stasis in one female dosed with 2.5×10^{12} GC/kg. Among male mice that received the highest vector dose, one had minimal hepatocellular mitotic figures and two had bile stasis. We did not detect liver abnormalities in female mice administered with the vehicle control; in males from the control group, we found only minimal centrilobular single-cell hepatocellular necrosis and/or degeneration and one mouse with intracanalicular bile stasis (Table 1).

We only reviewed non-liver tissues for the highest dose (2.5×10^{13} GC/kg) and the vehicle control groups (Table S2). In the lung, we observed minimal and mild accumulation of acidophilic material in alveolar macrophages in two males and one female administered with the vehicle control. Findings in the uterus consisted of minimal focal subacute inflammation in one female administered with the vehicle control. The skin on the injection site showed mild dermal fibrosis in one female administered with the highest vector dose.

Liver Vector Genome Copy and Transgene RNA Analysis

At the time of necropsy, we snap froze the liver and extracted DNA and RNA to quantify the vector GC and h*UGT1A1* transcript levels, respectively. Both vector GC (Figure 3A) and RNA levels (Figure 3B) increased in a dose-dependent manner, with no detectable levels in the vehicle control mice. The presence or absence of a statistical difference between the sexes administered with the same vector dose was consistent across vector GC and RNA levels, with the exception of mice dosed with 2.5×10^{10} or 2.5×10^{12} GC/kg. Male mice dosed at 2.5×10^{10} GC/kg showed significantly higher h*UGT1A1* RNA levels than females that received the same dose ($p < 0.01$, Mann-Whitney test). We observed a correlation between h*UGT1A1* RNA levels and serum total bilirubin levels among mice administered with the 2.5×10^{11} GC/kg dose. More specifically, we observed higher bilirubin levels on day 56 occurring in mice with lower levels of h*UGT1A1* RNA.

hUGT1A1 Expression in the UGT1 KO Mouse Liver

We collected liver samples at necropsy to measure hUGT1A1 expression at both mRNA and protein levels. We performed ViewRNA *in situ* hybridization (ISH) to detect h*UGT1A1*co mRNA expression and immunohistochemistry to evaluate protein expression (Figure 4). To avoid cross-hybridization with any potential endogenous mouse RNA from the gene locus, we used an ISH probe that is specific to the codon-optimized h*UGT1A1* sequence. Whereas ISH and immunohistochemistry staining increased with vector dose in female mice, male mice that were given the highest vector dose showed a slight reduction in staining. Although quantification of the ISH images confirmed this sex-specific observation, the difference was not

Table 1. Liver Histopathology Scoring

Dose (GC/kg)	2.5×10^{10}		2.5×10^{11}		2.5×10^{12}		2.5×10^{13}		Vehicle Control	
Sex	M	F	M	F	M	F	M	F	M	F
Single-cell hepatocellular necrosis and/or degeneration, centrilobular										
Minimal	2/5	2/5	1/5	4/5	3/5	2/5	1/5	1/5	1/5	–
Mild	–	–	–	–	–	–	2/5	–	–	–
Mononuclear cell infiltration										
Minimal	–	1/5	1/5	5/5	–	2/5	3/5	1/5	–	–
Mild	–	1/5	–	–	–	–	–	–	–	–
Mitotic figures, hepatocellular										
Minimal	–	–	–	1/5	–	1/5	1/5	–	–	–
Bile stasis										
Minimal	–	–	–	–	–	1/5	2/5	–	1/5	–

Male and female UGT1 KO mice received an i.v. injection of 2.5×10^{10}, 2.5×10^{11}, 2.5×10^{12}, or 2.5×10^{13} GC/kg of AAV8.TBG.hUGT1A1co or vehicle control (n = 5/sex/group). Liver sections positive for histopathology were scored as minimal, mild, moderate, marked, or severe. M, male; F, female.

statistically significant (Figure 4B). A stepwise increase of the dose by 10-fold did not result in significantly increased transgene expression, with the exception of male mice administered with 2.5×10^{11} and 2.5×10^{12} GC/kg that showed a statistically significant increase (Figure 4B).

DISCUSSION

We took several factors into consideration while designing this study. We chose to conduct the experiments in UGT1 KO mice, rather than wild-type mice or Gunn rats, for two reasons. First, we wanted to evaluate vector-associated toxicity within the setting of any pathology associated with the defect in UGT1A1, the associated hyperbilirubinemia, and its sequelae. Although we did not expect any liver pathology in the model, we were concerned that some level of chronic severe hyperbilirubinemia could influence the response of the host liver to the vector. Second, although others have previously evaluated adeno-associated viral (AAV) gene therapy for Crigler-Najjar syndrome in the classical animal model, the Gunn rat, this model does not require phototherapy treatment to survive to adulthood.[5,11–13] In a short-term study, i.v. administration with 5×10^{11} vector genomes (vg)/kg corrected total bilirubin levels in Gunn rats, and in a long-term study, seven of ten Gunn rats showed improvement following a dose of 5×10^{12} vg/kg.[13] The transient affect seen in three male rats was attributed to growth of the animal following vector administration at 6–8 weeks of age. Therefore, for the determination of the MED, we chose to use the more severe mouse model of Crigler-Najjar syndrome, the UGT1 KO mouse. Similar vector doses have been evaluated previously by others in $Ugt1^{-/-}$ mice, with correction of total bilirubin levels seen following intraperitoneal injection of 8×10^9 vg/mouse at post-natal day (P) 11[13] (which equates to a dose of $\sim 1.6 \times 10^{12}$ vg/kg, assuming a P11 animal weighs ~5 g[14]). Although this dose is approximately 10-fold higher than the MED reported here, the route of administration and age at dosing have to be taken into consideration when comparing efficacy.

In this study, male and female UGT1 KO mice aged 6–20 weeks received an i.v. injection of our clinical candidate vector, AAV8.TBG.hUGT1A1co, at one of four doses: 2.5×10^{10}, 2.5×10^{11}, 2.5×10^{12}, or 2.5×10^{13} GC/kg. We chose these doses to reflect the span of the proposed dosing regimen in a clinical trial. An additional cohort of animals received vehicle control. We collected blood samples from animals during the in-life phase to capture serum total bilirubin levels. On day 56 after administration, we necropsied the mice and harvested tissues for a comprehensive histopathological examination. We did not observe any clinical sequelae during the study.

Although we observed histopathological findings in male mice administered with the vehicle control, most findings were in male mice administered with the highest vector dose. All findings were minimal to mild, and microscopic findings were predominantly in the liver, characterized by centrilobular single-cell hepatocellular necrosis and mononuclear cell infiltration. In addition, we observed hepatocellular mitotic figures and minimal bile stasis. However, the liver findings occurred at all dose levels and in one vehicle control mouse. As the severity of the findings in the liver was consistent between the control- and the vector-administered groups, these can be considered background findings in the UGT1 KO mouse. Therefore, we observed no dose-limiting toxicities, and we can conclude that the maximally tolerated dose based on this mouse model study was greater than or equal to the highest dose tested (2.5×10^{13} GC/kg).

We observed a dose-dependent increase in liver transaminases; however, there was only a significant difference in ALT and AST following administration of the highest dose of vector (2.5×10^{13} GC/kg). The peak ALT and AST values occurred at day 28 and resolved by the end of the study. Although we did not evaluate immune responses to the AAV8 capsid, the hUGT1A1 transgene, or antibodies to hUGT1A1, there was no evidence of loss of transgene expression indicative of an adaptive immune response in these mice. In mice administered

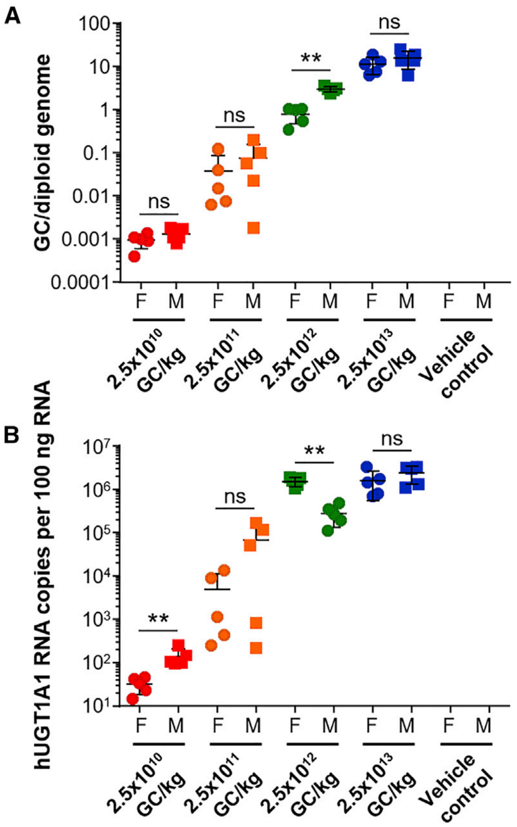

Figure 3. Liver Vector Biodistribution and h*UGT1A1* RNA Transcript Levels in Vector- or Vehicle Control-Injected UGT1 KO Mice

Male and female UGT1 KO mice received an i.v. injection of 2.5×10^{10}, 2.5×10^{11}, 2.5×10^{12}, or 2.5×10^{13} GC/kg of AAV8.TBG.hUGT1A1co or vehicle control. At necropsy, livers were harvested and snap frozen. (A) DNA was extracted for quantification of vector GC. (B) RNA was extracted for quantification of h*UGT1A1* transcript levels. Values are plotted as mean ± SD. NS, not significant. $^{**}p < 0.01$.

with 2.5×10^{13} GC/kg of vector, we did not observe a rebound in serum total bilirubin levels. Furthermore, liver vector genomes and hUGT1A1 RNA levels determined after necropsy on day 56 increased in a dose-dependent manner; we observed a slight reduction in ISH staining in male mice administered with the highest vector dose, but this difference was not statistically significant. In addition, the finding of minimal or mild mononuclear cell infiltration in this group by histopathological analysis was the same as in several vector-administered groups.

Following vector administration at doses of 2.5×10^{12} and 2.5×10^{13} GC/kg, serum total bilirubin levels rapidly and completely reversed to baseline levels of 0.1–0.3 mg/dL by day 14 after vector administration. At vector doses greater than 2.5×10^{10} GC/kg, total bilirubin levels were significantly reduced ($p < 0.05$). However, at the lowest dose (2.5×10^{10} GC/kg), the reduction in total bilirubin levels was temporary in male mice, and serum total bilirubin levels returned to baseline hyperbilirubinemia by day 42. At a dose of 2.5×10^{11} GC/kg, we noticed some variation in the response across five mice of each sex. Two males and three females showed a transient decrease in total bilirubin, with levels rising after day 14 after vector administration.

The transient serum total bilirubin levels following administration of 2.5×10^{11} GC/kg in both males and females could potentially allude to a threshold effect at this vector dose, such that a dose of 2.5×10^{11} GC/kg is effective in some animals, but not others. Again, because four of the five mice with transient expression also showed a concurrent rise in ALT, peaking at day 28, and reduced vector GC and RNA levels compared to other mice in the group, this effect could be due to an immune response to the vector or human transgene. We did not harvest splenocytes at the time of necropsy to determine the presence of a cytotoxic T lymphocyte response. Although these mice had minimal histopathology findings, the findings were not enhanced compared to other mice in the group or in other vector dose groups. Transient effects of gene therapy are commonly seen following newborn administration due to rapid growth of the animal.[4,8,10,14] However, it is unlikely that the transient effect seen here was due to growth of the mice during the study, because the average body weight only increased from 18.8 ± 2.5 to 22.0 ± 2.2 g (mean ± SD) over the two-month study.

Administration of the lowest vector dose (2.5×10^{10} GC/kg) in female UGT1 KO mice did not result in deviation from baseline total bilirubin values; however, there was a 79% reduction in total bilirubin in male mice at the same dose. In addition, although there was no significant difference in liver vector GCs between male and female mice administered with 2.5×10^{10} GC/kg of vector, male mice showed 4-fold higher hUGT1A1 RNA levels compared to females. This indicates that gene transfer to hepatocytes was similar but that the higher transgene RNA levels in males was sufficient to be transiently therapeutic. Other researchers have reported a sex difference in both transgene expression and vector GC following vector dosing in mice.[9,15] Following i.v. administration of 3.3×10^{11} vg/mouse (calculated by the authors to be $\sim 1.1 \times 10^{13}$ vg/kg) at P60 in Ugt1$^{-/-}$ mice, male mice showed a correction of total bilirubin to wild-type levels. However, female mice showed a transient reduction in total bilirubin levels with a significant reduction in vector genomes.[9] This sex difference does not appear to translate to non-human primates.

Based on the significant reduction in serum total bilirubin levels in mice following vector administration, the MED in this mouse model of Crigler-Najjar syndrome is 2.5×10^{11} GC/kg. The dose required for sustained and complete normalization of total bilirubin levels was 2.5×10^{12} GC/kg; however, because the doses evaluated here increased by one log, it is possible that a dose between 2.5×10^{11} and 2.5×10^{12} GC/kg will also achieve this. The MED corresponds to approximately 1.9% and 1.2% transduction of hepatocytes in female and male mice, respectively, based on ISH data (Figure 4B). This is the same percentage of liver transduction in adult mice that is retained following newborn systemic vector administration and was sufficient to rescue UGT1 KO mice from lethal hyperbilirubinemia.[10]

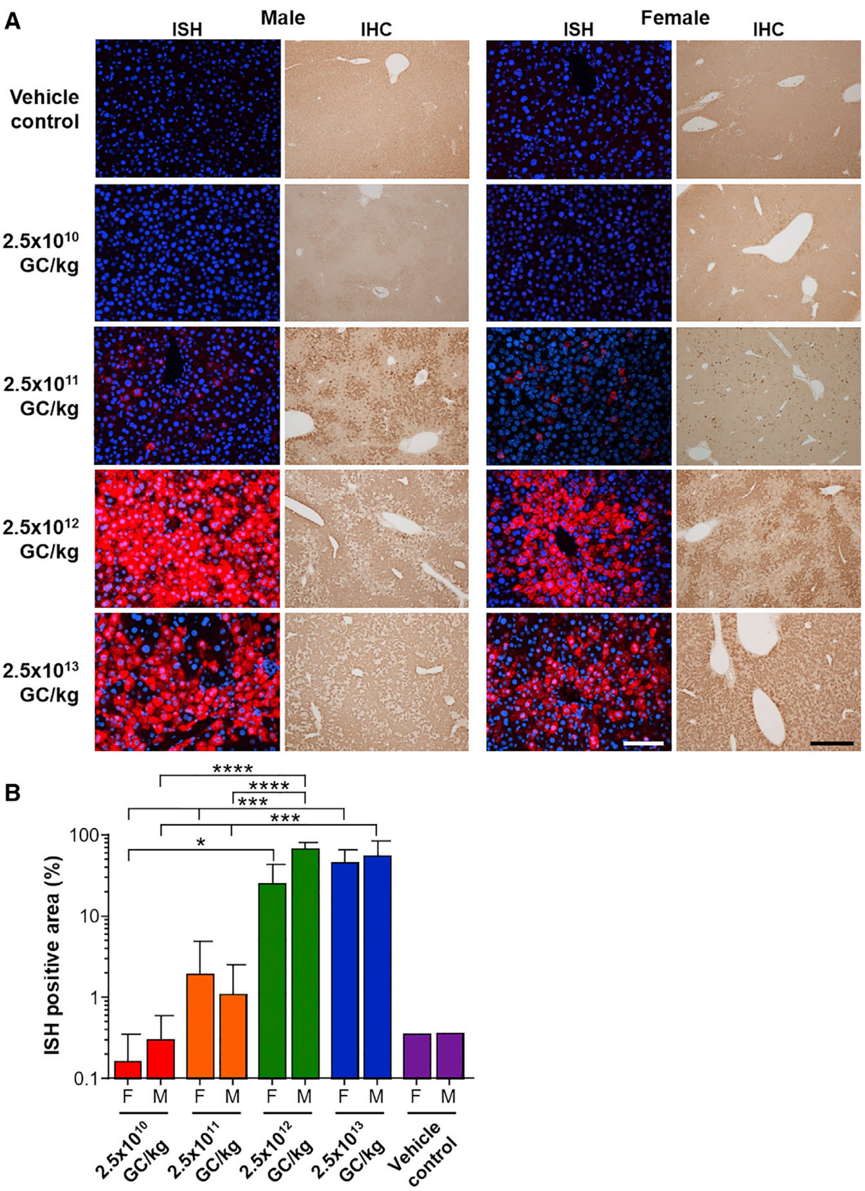

Figure 4. hUGT1A1 Expression in Liver of Vector-Injected UGT1 KO Mice

Male and female UGT1 KO mice received an i.v. injection of 2.5×10^{10}, 2.5×10^{11}, 2.5×10^{12}, or 2.5×10^{13} GC/kg of AAV8.TBG.hUGT1A1co or vehicle control. (A) ISH and immunohistochemistry (IHC) staining were performed on liver sections for detection of hUGT1A1. In ISH sections, h*UGT1A1*co RNA is shown with red staining, and sections were counterstained with the nuclear stain DAPI (blue). Representative images are from mice with stable serum total bilirubin levels. Scale bars: 100 μm for ISH, 500 μm for IHC. (B) ISH staining was quantified, and the percentage of positively stained area was calculated. Values are expressed as mean ± SD. *$p < 0.05$, ***$p < 0.001$, ****$p < 0.0001$.

MATERIALS AND METHODS

AAV Vector Production

All AAV vectors were produced by the Penn Vector Core at the University of Pennsylvania, as described previously.[16] Plasmids expressing a codon-optimized version of hUGT1A1 from the TBG promoter were packaged with the AAV8 viral capsid to create the single-stranded vector, AAV8.TBG.hUGT1A1co.

Mice

Breeding pairs of heterozygous Ugt1$^{+/-}$ mice (mixed B6 and 129 background strains) were obtained from The Jackson Laboratory (Bar Harbor, ME), and a colony was maintained at the University of Pennsylvania under specific pathogen-free conditions. All mice used for this study were derived from this colony. All animal procedures and protocols were approved by the Institutional Animal Care and Use Committee of the University of Pennsylvania. Mice were individually housed in disposable micro-isolator mouse caging with corncob bedding and Nestlets provided for enrichment (Innovive, San Diego, CA). Certified irradiated Laboratory Rodent Diet 5002 (LabDiet, St. Louis, MO) was provided *ad libitum*. All interventions were performed during the light cycle, and mice were not fasted before blood collection.

Male and female UGT1 KO mice (n = 51, 26 male and 25 female) 6 to 20 weeks in age and greater than 15 g in body weight were used in this study and necropsied at day 56 after administration (Table S1). Group dosing dates were staggered based on availability of UGT1 KO mice. At the initiation of the study, available mice within the dosing age range were randomly assigned first to the high-dose group (2.5×10^{13} GC/kg) or to the control group by an online program (Research Randomizer, http://www.randomizer.org/). Subsequently, mice were assigned to dose groups in the following order: 2.5×10^{12}, 2.5×10^{10}, and 2.5×10^{11} GC/kg.

Serum Analyses

Blood was collected every other week and allowed to clot. Serum was isolated and sent to Antech GLP (Morrisville, NC) for analysis of ALT, AST, and total bilirubin levels.

Histopathology

At day 56 after administration, mice were euthanized. Blood was collected by cardiac puncture, and a full necropsy was performed. Tissues were harvested for comprehensive histopathological examination (see Table S3 for the list of harvested tissues). Tissues were fixed

using 10% neutral buffered formalin, paraffin embedded, sectioned, and stained for histopathology using H&E stain. An experienced board-certified veterinary pathologist microscopically examined all tissues from the highest vector dose group and the vehicle control group in a blinded manner. Liver sections were evaluated for histopathology from all dose groups in the same manner.

Immunohistochemistry

Liver sections were deparaffinized through an ethanol and xylene series, boiled for 6 min in 10 mM citrate buffer (pH 6.0) for antigen retrieval, and sequentially treated with 2% H_2O_2 (15 min), avidin and biotin blocking reagents (15 min each; Vector Laboratories, Burlingame, CA), and blocking buffer (1% donkey serum in PBS with 0.2% Triton for 10 min). Sections were incubated with 5 μg/mL of primary antibody against UGT1A1 (R&D Systems, Minneapolis, MN) for 1 hr and then with a biotinylated secondary antibody (Jackson ImmunoResearch, West Grove, PA) diluted in blocking buffer for 45 min. A Vectastain Elite ABC Kit (Vector Laboratories) was used according to the manufacturer's instructions with 3,3′-diaminobenzidine as the substrate to stain bound antibodies as a brown precipitate.

In Situ Hybridization

ISH was performed on formalin-fixed, paraffin-embedded liver sections using the ViewRNA ISH Tissue Assay Kit (Thermo Fisher Scientific, Waltham, MA) according to the manufacturer's instructions. Z-shaped probe pairs specific for hUGT1A1co were synthesized by the kit manufacturer. The deposition of Fast Red Substrate (Thermo Fisher Scientific) precipitates indicating positive signals was imaged by fluorescence microscopy using a rhodamine filter set. Sections were counterstained with DAPI to visualize nuclei.

To quantify RNA expression, five random pictures were taken from each liver section. Using ImageJ software (https://imagej.nih.gov/ij/), a threshold was determined to select UGT1A1-ISH-positive areas, and the percentage of positive area per image area was established. Likewise, an empty liver area (i.e., veins) was quantified in a second measurement. The final percentage of ISH-positive liver tissue (i.e., the percentage of positive hepatocytes) was calculated as the adjusted area (i.e., total area minus empty area), and the values were averaged for each liver.

Vector Genome Copy and Transgene RNA Analysis

Liver was snap frozen at the time of necropsy, and DNA was extracted using the QIAamp DNA Mini Kit (QIAGEN, Valencia, CA). Detection and quantification of vector GC in extracted DNA and relative hUGT1A1 transcript expression in extracted RNA were performed by real-time PCR, as described previously.[17,18] Vector GC and RNA levels were quantified using primers and probe designed against the poly(A) sequence of the vector and a transgene-specific sequence, respectively.

Statistical Analysis

We calculated and reported the cohort average and SD for ALT, AST, total bilirubin, vector GC, and h*UGT1A1* RNA transcript data. Differences in ALT, AST, and total bilirubin levels compared to baseline were analyzed statistically by the Wilcoxon rank-sum test, and overall differences in ALT, AST, and total bilirubin values across all time points were analyzed using a linear mixed-effect model. Analyses were stratified by sex. Comparisons between two groups were performed using a non-parametric Mann-Whitney test, and comparisons among multiple groups were performed using a one-way ANOVA (Tukey's multiple comparison post-test). A p value < 0.05 was considered significant.

SUPPLEMENTAL INFORMATION

Supplemental Information includes three tables and can be found with this article online at https://doi.org/10.1016/j.omtm.2018.07.008.

AUTHOR CONTRIBUTIONS

Conceptualization, J.A.G., J.T.G., and J.M.W.; Methodology, J.A.G. and J.M.W.; Validation, E.A.C.; Investigation, J.A.G., J.M.L.N., C.D., D.M., P.B., and L.K.R.; Writing – Original Draft, J.A.G.; Writing – Review & Editing, J.A.G., J.T.G., and J.M.W.; Visualization, J.A.G. and J.M.W.; Supervision, J.A.G., J.T.G., and J.M.W.; Funding Acquisition, J.M.W.

CONFLICTS OF INTEREST

J.M.W. is an advisor to, holds equity in, and has a sponsored research agreement with REGENXBIO and Scout Bio; he also has sponsored research agreements with Ultragenyx, Biogen, and Janssen, which are licensees of Penn technology. In addition, he has sponsored research agreements with Precision Biosciences and Moderna Therapeutics. J.M.W. holds equity in Solid Bio, and he is an inventor on patents that have been licensed to various biopharmaceutical companies.

ACKNOWLEDGMENTS

We would like to thank Melanie Smith and Yanqing Zhu for invaluable technical assistance, as well as Mingyao Li and Yan Che for help with statistical analysis. This research was supported by Audentes Therapeutics.

REFERENCES

1. Chen, S.J., Sanmiguel, J., Lock, M., McMenamin, D., Draper, C., Limberis, M.P., Kassim, S.H., Somanathan, S., Bell, P., Johnston, J.C., et al. (2013). Biodistribution of AAV8 vectors expressing human low-density lipoprotein receptor in a mouse model of homozygous familial hypercholesterolemia. Hum. Gene Ther. Clin. Dev. *24*, 154–160.

2. Greig, J.A., Limberis, M.P., Bell, P., Chen, S.J., Calcedo, R., Rader, D.J., and Wilson, J.M. (2017). Nonclinical pharmacology/toxicology study of AAV8.TBG.mLDLR and AAV8.TBG.hLDLR in a mouse model of homozygous familial hypercholesterolemia. Hum. Gene Ther. Clin. Dev. *28*, 28–38.

3. Nguyen, N., Bonzo, J.A., Chen, S., Chouinard, S., Kelner, M.J., Hardiman, G., Bélanger, A., and Tukey, R.H. (2008). Disruption of the ugt1 locus in mice resembles human Crigler-Najjar type I disease. J. Biol. Chem. *283*, 7901–7911.

4. Bortolussi, G., Zentilin, L., Baj, G., Giraudi, P., Bellarosa, C., Giacca, M., Tiribelli, C., and Muro, A.F. (2012). Rescue of bilirubin-induced neonatal lethality in a mouse model of Crigler-Najjar syndrome type I by AAV9-mediated gene transfer. FASEB J. *26*, 1052–1063.

5. Gunn, C.K. (1944). Hereditary Acholuric Jaundice in the Rat. Can. Med. Assoc. J. *50*, 230–237.

6. Iyanagi, T., Watanabe, T., and Uchiyama, Y. (1989). The 3-methylcholanthrene-inducible UDP-glucuronosyltransferase deficiency in the hyperbilirubinemic rat (Gunn rat) is caused by a −1 frameshift mutation. J. Biol. Chem. *264*, 21302–21307.

7. Bortolussi, G., Baj, G., Vodret, S., Viviani, G., Bittolo, T., and Muro, A.F. (2014). Age-dependent pattern of cerebellar susceptibility to bilirubin neurotoxicity *in vivo* in mice. Dis. Model. Mech. *7*, 1057–1068.

8. Bortolussi, G., Zentillin, L., Vaníkova, J., Bockor, L., Bellarosa, C., Mancarella, A., Vianello, E., Tiribelli, C., Giacca, M., Vitek, L., and Muro, A.F. (2014). Life-long correction of hyperbilirubinemia with a neonatal liver-specific AAV-mediated gene transfer in a lethal mouse model of Crigler-Najjar syndrome. Hum. Gene Ther. *25*, 844–855.

9. Bočkor, L., Bortolussi, G., Iaconcig, A., Chiaruttini, G., Tiribelli, C., Giacca, M., Benvenuti, F., Zentilin, L., and Muro, A.F. (2017). Repeated AAV-mediated gene transfer by serotype switching enables long-lasting therapeutic levels of hUgt1a1 enzyme in a mouse model of Crigler-Najjar syndrome type I. Gene Ther. *24*, 649–660.

10. Greig, J.A., Nordin, J.M.L., Draper, C., Bell, P., and Wilson, J.M. (2018). AAV8 gene therapy rescues the newborn phenotype of a mouse model of Crigler-Najjar. Hum. Gene Ther. *29*, 763–770.

11. Seppen, J., Bakker, C., de Jong, B., Kunne, C., van den Oever, K., Vandenberghe, K., de Waart, R., Twisk, J., and Bosma, P. (2006). Adeno-associated virus vector serotypes mediate sustained correction of bilirubin UDP glucuronosyltransferase deficiency in rats. Mol. Ther. *13*, 1085–1092.

12. Montenegro-Miranda, P.S., Pañeda, A., ten Bloemendaal, L., Duijst, S., de Waart, D.R., Gonzalez-Aseguinolaza, G., and Bosma, P.J. (2013). Adeno-associated viral vector serotype 5 poorly transduces liver in rat models. PLoS ONE *8*, e82597.

13. Ronzitti, G., Bortolussi, G., van Dijk, R., Collaud, F., Charles, S., Leborgne, C., Vidal, P., Martin, S., Gjata, B., Sola, M.S., et al. (2016). A translationally optimized AAV-UGT1A1 vector drives safe and long-lasting correction of Crigler-Najjar syndrome. Mol. Ther. Methods Clin. Dev. *3*, 16049.

14. Wang, L., Wang, H., Bell, P., McMenamin, D., and Wilson, J.M. (2012). Hepatic gene transfer in neonatal mice by adeno-associated virus serotype 8 vector. Hum. Gene Ther. *23*, 533–539.

15. Davidoff, A.M., Ng, C.Y., Zhou, J., Spence, Y., and Nathwani, A.C. (2003). Sex significantly influences transduction of murine liver by recombinant adeno-associated viral vectors through an androgen-dependent pathway. Blood *102*, 480–488.

16. Gao, G., Lu, Y., Calcedo, R., Grant, R.L., Bell, P., Wang, L., Figueredo, J., Lock, M., and Wilson, J.M. (2006). Biology of AAV serotype vectors in liver-directed gene transfer to nonhuman primates. Mol. Ther. *13*, 77–87.

17. Bell, P., Moscioni, A.D., McCarter, R.J., Wu, D., Gao, G., Hoang, A., Sanmiguel, J.C., Sun, X., Wivel, N.A., Raper, S.E., et al. (2006). Analysis of tumors arising in male B6C3F1 mice with and without AAV vector delivery to liver. Mol. Ther. *14*, 34–44.

18. Greig, J.A., Peng, H., Ohlstein, J., Medina-Jaszek, C.A., Ahonkhai, O., Mentzinger, A., Grant, R.L., Roy, S., Chen, S.J., Bell, P., et al. (2014). Intramuscular injection of AAV8 in mice and macaques is associated with substantial hepatic targeting and transgene expression. PLoS ONE *9*, e112268.

OMTM, Volume *10*

Supplemental Information

Determining the Minimally Effective Dose of a Clinical Candidate AAV Vector in a Mouse Model of Crigler-Najjar Syndrome

Jenny A. Greig, Jayme M.L. Nordin, Christine Draper, Deirdre McMenamin, Edward A. Chroscinski, Peter Bell, John T. Gray, Laura K. Richman, and James M. Wilson

Supplemental Table 1. Composition of study groups.

Dose	Female		Male	
	Mouse	Age (weeks)	Mouse ID	Age (weeks)
$2.5x10^{10}$ GC/kg	5228	8	4093	12
	6944	11	4913	8
	7524	6	4971	7
	6851	11	5018	7
	6678	15	5020	7
$2.5x10^{11}$ GC/kg	7426	7	5147	7
	6311	18	5149	7
	6753	13	5182	9
	6211	20	5264	7
	7442	7	6989	9
$2.5x10^{12}$ GC/kg	5054	8	4161	7
	5127	7	4162	7
	5128	7	4178	7
	5129	7	4553	6
	5198	9	4573	6
$2.5x10^{13}$ GC/kg	4158	6	4094	7
	4159	6	2786	13
	988	11	2764	15
	2782	13	2717	15
	2374	19	4576	6
Vehicle control	983	11	2785	13
	2369	19	2716	15
	2375	19	990	11
	4044	8	2718	15
	4549	6	945	11

Supplemental Table 2. Histopathology summary from vector- and vehicle control-injected UGT1 KO mice.

**Cohort**	**1**	**1**	**2**	**2**	**3**	**3**	**4**	**4**	**5**	**5**
**Sex**	**M**	**F**	**M**	**F**	**F**	**M**	**F**	**M**	**F**	**M**
**No. of animals evaluated per cohort**	**5***	**5***	**6***	**5***	**5***	**5***	**5**	**5**	**5**	**5**
**Liver**										
No. examined	5	5	6	5	5	5	5	5	5	5
No abnormalities detected	3	2	5	-	3	2	4	2	5	4
	---	---	---	---	---	---	---	---	---	---
Single cell hepatocellular necrosis/degeneration, centrilobular										
Grade 1	2	2	1	4	2	3	1	1	-	1
Grade 2	-	-	-	-	-	-	-	2	-	-
	---	---	---	---	---	---	---	---	---	---
Mononuclear cell infiltration										
Grade 1	-	1	1	5	2	-	1	3	-	-
Grade 2	-	1	-	-	-	-	-	-	-	-
	---	---	---	---	---	---	---	---	---	---
Mitotic figures, hepatocellular										
Grade 1	-	-	-	1	1	-	-	1	-	-
	---	---	---	---	---	---	---	---	---	---
Bile stasis										
Grade 1	-	-	-	-	1	-	-	2	-	1
**Lung**										
No. examined	-	-	-	-	-	-	5	5	5	5
No abnormalities detected	-	-	-	-	-	-	5	5	3	3
	---	---	---	---	---	---	---	---	---	---
Alveolar macrophages, acidophilic material accumulation										
Grade 1	-	-	-	-	-	-	-	-	1	1
Grade 2	-	-	-	-	-	-	-	-	-	1
	---	---	---	---	---	---	---	---	---	---
Infiltrate, subacute, focal										
Grade 1	-	-	-	-	-	-	-	-	1	-
**Uterus**										
No. examined	-	-	-	-	-	-	5	-	5	-
No abnormalities detected	-	-	-	-	-	-	5	-	4	-
	---	---	---	---	---	---	---	---	---	---
Inflammation, subacute, focal										
Grade 1	-	-	-	-	-	-	-	-	1	-
**Skin, injection site**										
No. examined	-	-	-	-	-	-	5	5	5	5
No abnormalities detected	-	-	-	-	-	-	4	5	5	5
	---	---	---	---	---	---	---	---	---	---
Dermal fibrosis										
Grade 2	-	-	-	-	-	-	1	-	-	-

Supplemental Table 3. Tissues harvested for histopathology.

Tissues harvested for histopathology		
Adrenal glands	Jejunum	Skeletal muscle
Brain	Kidneys	Spleen
Cecum	Liver	Sternum
Colon	Lungs	Stomach
Duodenum	Mandibular lymph node	Testes
Epididymides	Mesenteric lymph node	Thymus
Eyes	Ovaries	Thyroids with parathyroids
Heart	Pancreas	Urinary bladder
Ileum	Salivary glands	Uterus
Injection site	Sciatic nerve	Gross lesions (if any)
